# Advanced Dynamic Centre of Pressure Diagnostics with Smart Insoles: Comparison of Diabetic and Healthy Persons for Diagnosing Diabetic Peripheral Neuropathy

**DOI:** 10.3390/bioengineering11121241

**Published:** 2024-12-08

**Authors:** Franz Konstantin Fuss, Adin Ming Tan, Yehuda Weizman

**Affiliations:** 1 Chair of Biomechanics, Faculty of Engineering Science, University of Bayreuth, D-95440 Bayreuth, Germany; yehuda.weizman@uni-bayreuth.de (Y.W.); adintan@gmail.com (A.M.T.); 2 Division of Biomechatronics, Fraunhofer Institute for Manufacturing Engineering and Automation IPA, D-95447 Bayreuth, Germany; 3 Faculty of Health, Arts and Design, Swinburne University, Melbourne, VIC 3000, Australia

**Keywords:** cyclogram, butterfly diagram, wearable technology, classifier, receiver operating characteristic curve, AUC, sensitivity, specificity, balance index, pressure sensitive insole

## Abstract

Although diabetic polyneuropathy (DPN) has a very high prevalence among people with diabetes, gait analysis using cyclograms is very limited, and cyclogram research, in general, is limited to standard measures available in software packages. In this study, cyclograms (movements of the centre of pressure, COP, on and between the plantar surfaces) of diabetics and healthy individuals recorded with a smart insole were compared in terms of geometry and balance index, BI. The latter was calculated as the summed product of standard deviations of cyclogram markers, i.e., start/end points, turning points, and intersection points of the COP. The geometry was assessed by the positions of, and distances between, these points, and the distance ratios (14 parameters in total). The BI of healthy and diabetic individuals differed significantly. Of the fifteen parameters (including the BI), three were suitable as classifiers to predict DPN, namely two distances and their ratio, with false negatives ranging from 1.8 to 12.5%, and false positives ranging from 2.9 to 7.1%. The standard metric of the cyclogram provided by the software packages failed as a classifier. While the BI captures both DPN-related balance and other balance disorders, the changing geometry of the cyclogram in diabetics appears to be DPN-specific.

## 1. Introduction

The International Diabetes Federation [1] “*estimated that in 2017 there are 451 million … people*”, i.e., one in every eleven people, with diabetes worldwide. “*In 2016, there were an estimated 131.0 million people (1.77% of the global population) affected by overall*” diabetes-related lower-extremity complications, “*including 105.6 million … with neuropathy only*” and “*18.6 million … with foot ulcers…*” [2]. Diabetic peripheral neuropathy (DPN) is the causative agent for the development of foot ulcers and amputations [3]. In 1993, the prevalence of DPN for type 1 diabetes (T1D) and type 2 diabetes (T2D) combined increased from 35% to 68% across an age of 60–90 years [4]. In 2020, the prevalence of DPN for T1D and T2D increased from 36.1% and 38.8% to 60% and 52.4%, respectively, for an age of 56 to >70 years [5].

DPN includes sensory, motor, and autonomic neuropathy [6]. DPN is diagnosed by assessing the function of the large and small myelinated nerve fibres [7]. The latter are tested for thermal discrimination and pinprick sensations; the large fibres are tested with the standard Semmes–Weinstein 5.07 monofilament 10 g of pressure protective sensation test, vibration perception test, and ankle reflexes tests [6,7].

Regarding motor neuropathy, Pop-Busui et al. [7] only mention “poor balance” as a symptom of DPN, but do not propose any standard balance test, likely due to the high cost of the necessary equipment.

Balance can be assessed statically and dynamically. The static test is performed on a posturographic system or on a simple 3D force plate and measures the scatter of the centre of pressure (COP). The cyclogram or butterfly diagram, which measures the COP movement between both feet during walking, is the standard dynamic test. Devices that can perform a dynamic test are either stationary (treadmills and instrumented walkways) or mobile, such as wearable technology (instrumented smart insoles or shoes).

**Figure 1:**
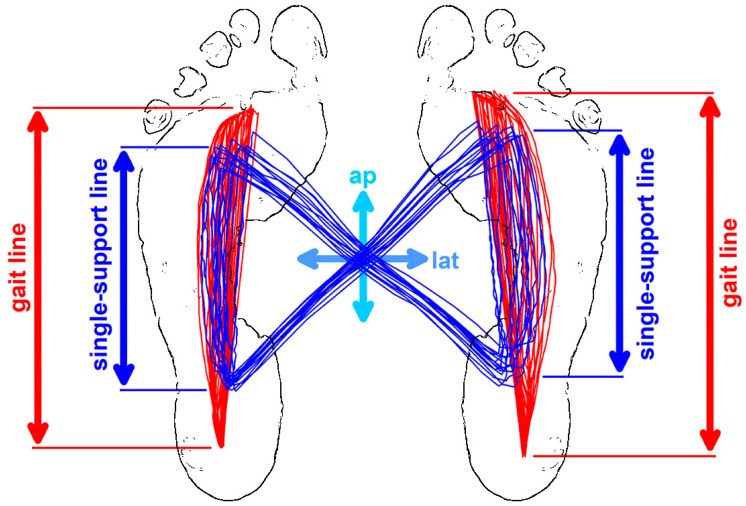
Standard COP measurements; outline of the cyclogram (blue), projected on the COP paths of the left and right foot (red); the gait line corresponds to the total COP movement underneath each foot during the entire stance phase; the single support line is the COP movement in the single-support phase; the diagonals of the cyclogram represent the COP movement in the double-support phase; ap: antero-posterior position and variability of the COP intersection points; lat: lateral position and variability of the COP intersection points.

Automated data analysis is often implemented in software packages of devices (e.g., Zebris [8], Noraxon [9], and Medilogic [10]) that measure the COP using some or all of the following six measurements of the cyclogram (or butterfly diagram; Figure 1):

- Length of gait line;
- Length of single-support line;
- Antero-posterior position of the COP intersection;
- Lateral shift of the COP position of the COP intersection;
- Antero-posterior variability (standard deviation of the COP intersections); and
- Lateral variability (standard deviation of the COP intersections).

Table 1 lists the state of the art of cyclogram applications [11,12,13,14,15,16,17,18,19,20,21,22,23,24,25,26,27,28,29] using different technologies (treadmills, instrumented walkways, and smart shoes or insoles). Most of the studies in Table 1 use one or more of the six standard measurements. In contrast, Wong et al. [22,23] defined cyclogram patterns as a specific metric, while Agrarwal et al. [26] used additional distances between the cyclogram markers.

Agrawal et al. [26] assessed fall risk in study participants aged above 65 years who had no medical conditions with a commercially available insole with five piezo-resistive sensors. They investigated several distances within the cyclogram using machine learning models for predicting fall risk, such as random forest (RF) and logistic regression (LR). The right and left single-support lines (standard measurement of the cyclogram) showed the highest feature scores among all distances investigated. The LR and RF models showed the highest AUCs of 0.82 (sensitivity 74%, specificity 76%) and 0.81 (sensitivity 56%, specificity 88%), respectively.

Kalron and Frid [12] also investigated classifiers to assess the difference between healthy people and patients with multiple sclerosis. When comparing cerebellar function in non/slight disability and mild/moderate disability, the ROC (receiver operating characteristic) curve of the COP variability in the antero-posterior direction had an AUC (area under the ROC curve) of 0.778, and sensitivity and specificity of 83.8% and 62.4%, respectively.

Surprisingly, the literature overview (Table 1) could only find one study that dealt with diabetes and butterfly diagrams. Cao et al. [27] compared diabetic patients with or without DPN or peripheral arterial disease (PAD) and healthy people by using only the antero-posterior position of the COP intersection point and concluded that the COP differed significantly between DPN and healthy conditions, while it was insignificant between DAP, diabetes without DAP, and healthy conditions. Cao et al. [27] did not use a classifier approach like Kalron and Frid [12] and Agrawal et al. [26], but calculated the AUC and sensitivity and specificity.

The six parameters provided by the software of the measuring devices listed in Table 1 limit the possibilities for innovation in the field of cyclogram diagnostics. Agrawal et al. [26] have already gone a step further and included some distances between the markers of the cyclogram. However, a thorough analysis of all possible distances and their ratios is missing in the literature. Such an analysis regarding the sensitivity and specificity of new parameters would enable the introduction of new diagnostic tools for cyclogram applications.

Therefore, the aim of this study was to evaluate further cyclogram-related parameters, in addition to the six standard measurements, and to test their performance in classifying subjects as diabetic people with DPN and as healthy people.

## 2. Materials and Methods

### 2.1. Participants

This study included 14 people with diabetes and peripheral neuropathy and 15 healthy volunteers (as in a previous study [30]).

The diabetics were recruited from the patient pool of the podiatry clinic at the Queensland University of Technology. The inclusion criteria were as follows: T2D, DPN, and shoe sizes 9 and 11 (due to the availability of the sizes of the measurement system). The exclusion criteria were as follows: open diabetic ulcer, Charcot foot, and walking only with assistance. The 14 patients (9 men and 5 women; age range 58–80 and mean 69 ± 11; age group with high DPN prevalence [4,5]) had been diagnosed with T2D at least 5 years previously, and all had been diagnosed with peripheral neuropathy (defined as at least loss of protective sensation measured with a 10 g monofilament by a registered podiatrist). A standardized pair of closed and laced shoes was worn during the walking trials.

The 15 healthy volunteers (14 men and 1 woman; age range 20–55, average 33 ± 9) of the control group were recruited from students and staff of the SportzEdge (Sports Engineering) Research Program at the School of Aerospace, Mechanical and Manufacturing Engineering via personal contacts. The inclusion criteria were as follows: shoe sizes 9 and 11. The exclusion criteria were as follows: diabetes, age > 60, lower extremity musculoskeletal disorders, gait impairments, pain in the legs and feet, and history of foot ulcers.

### 2.2. Experimental Procedure

We provided ASICS Gel 530TR shoes (sizes 9 and 11; ASICS Oceania, Marsden Park, Australia) to the two cohorts, fitted with Pedar pressure-sensitive insoles (99 sensors, sizes 9 and 11; Novel Inc., Munich, Germany). Three diabetic patients, however, had to wear their preferred pair of closed and laced shoes because of orthopedic insoles that did not fit into the standardized shoes. The Pedar insoles were calibrated with the *trublu* calibration device (Novel Inc., Munich, Germany) up to a pressure of 0.6 MPa.

All participants were equipped with a Pedar system (Novel Inc., Munich, Germany). They were asked to walk down a 15 m flat walkway at a comfortable pace. Data from each participant were collected from 12 consecutive steps [31] at a sampling frequency of 50 Hz. The Pedar insoles were calibrated before testing. The insole pressure data were collected at least twice for each participant and results were accepted only if the walking speeds of two datasets were within 10% of each other. Time was recorded with a stopwatch and distance was measured with a tape measure. Since the absolute and relative duration of the stance phase, swing phase, and double support are a function of walking speed and the influence of speed on gait indices (movement and distribution of the COP) is unknown, we wanted to ensure that these indices were not influenced by another independent variable (i.e., by the walking speed). The participants’ force–time curves were visually inspected and only steps that were considered regularly and continuously distributed were extracted from the dataset. The total number of datasets was 126.

### 2.3. Data Processing

**Figure 2:**
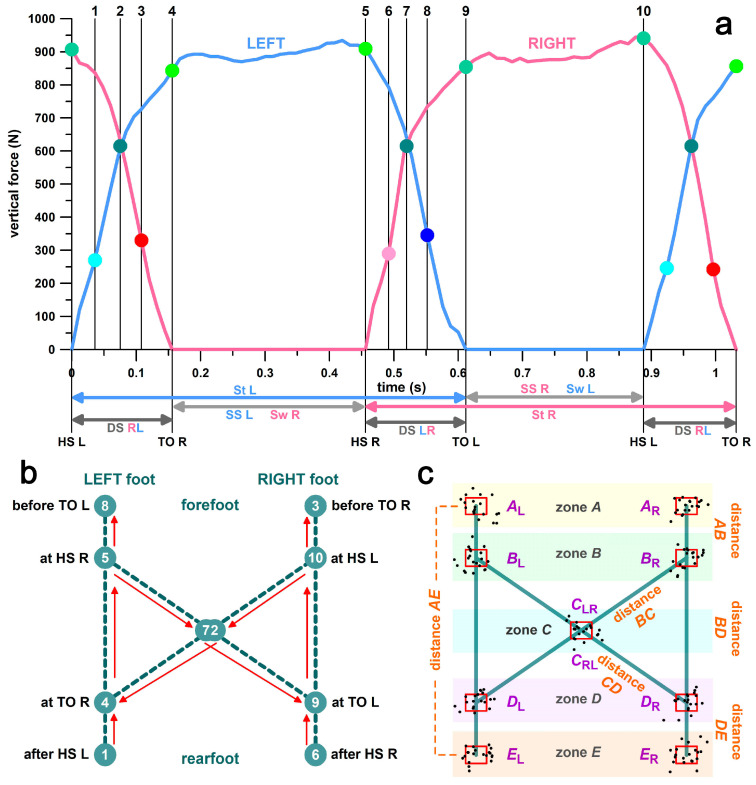
Explanation of timing and locations of the COP and the cyclogram; (**a**) vertical forces of a diabetic individual during one stride measured with the Pedar insoles; the numbers on the top (1–10) and the corresponding dots represent the time markers of the cyclogram; the corresponding phases of the gait cycle are indicated at the bottom; R = right; L = left; St = stance phase; SS = single-support phase; DS = double-support phase; HS = heel strike; TO = toe-off; (**b**) spatiotemporal diagram of the cyclogram; the numbers (1–10) correspond to the time markers shown in (**a**); the red arrows indicate the direction of COP movement; (**c**) 2D markers (*A*–*E*) and their distances; the black dots represent the scatter of the markers; the red rectangles are centred at the average of the scattered markers; the side lengths of the red rectangles correspond to 2 standard deviations of the scatter; note that the sum of distances *AB*, *BD*, and *DE* is not identical to *AE*, since these distances are not in line, as shown in (**a**,**c**) for simplicity reasons; distance *BD* = single-support line; diagonals (*B*_R_*D*_L_, *B*_L_*D*_R_) = double-support, distance *AE* = gait line (stance phase).

The force and COP data from the Pedar were extracted and a Matlab (R2021a) procedure (MathWorks, Inc., Natick, MA, USA) was written to identify the different phases of each gait cycle [30]. The COP data of right and left feet were converted to a cyclogram or butterfly diagram by calculating the weighted average of right and left x- and y-coordinates of the COP, weighted by the instantaneous forces of the right and left feet.

(1) $${COP}_{Cx}=\frac{F_{R}\;{COP}_{Rx}+F_{L}\;{COP}_{Lx}}{F_{R}+F_{L}}\;\;\;\&\;\;\;{COP}_{Cy}=\frac{F_{R}\;{COP}_{Ry}+F_{L}\;{COP}_{Ly}}{F_{R}+F_{L}}\;$$

where COP*_Cx_* and COP*_Cy_* are the COP coordinates of the cyclogram, COP*_R,Lx_* and COP*_R,Ly_* are the COP coordinates of the right and left feet, and *F_R,L_* are the forces of the right and left feet. All COP data were normalised to the sizes of the insoles. The following ten COP data points (x- and y-coordinates) were extracted for each step from the individual feet and the cyclogram (Figure 2):

- Rearmost COP positions after heel strike: points 1 and 6 in Figure 2a,b; points *E* in Figure 2c (COP_Lmin_ & COP_Rmin_).
- Frontmost COP-positions before toe-off: points 3 and 8 in Figure 2a,b; points *A* in Figure 2c (COP_Lmax_ & COP_Rmax_).
- COP positions at toe-off of the contralateral leg: points 4 and 9 in Figure 2a,b; points *D* in Figure 2c (COP**_C_**_Lmin_ & COP**_C_**_Rmin_).
- COP positions at heel strike of the contralateral leg: points 5 and 10 in Figure 2a,b; points *B* in Figure 2c (COP**_C_**_Lmax_ & COP**_C_**_Rmax_).
- Intersection points of COP trajectories in the centre of the double support, weight shifting from left to right or right to left: points 7 and 2 in Figure 2a,b; points *C* in Figure 2c.

The numbers from 1 to 10 in Figure 2a,b refer to the temporal sequence of the COP positions shown in Figure 2b. The spatiotemporal sequence of the COP positions in the left footprint is 1-4-5-8 in Figure 2b and *E*_L_-*D*_L_-*B*_L_-*A*_L_ in Figure 2c, and in the right footprint 6-9-10-3 in Figure 2b and *E*_R_-*D*_R_-*B*_R_-*A*_R_ in Figure 2c. The spatiotemporal sequence of the COP positions in the cyclogram is 4-5-7-9-10-2-4-, etc., in Figure 2b and *D*_L_-*B*_L_-*C*_LR_-*D*_R_-*B*_R_-*C*_RL_-*D*_L_-, etc., in Figure 2c (subscripts _LR_ and _RL_ denote the COP movements from left to right and right to left, respectively).

**Figure 3:**
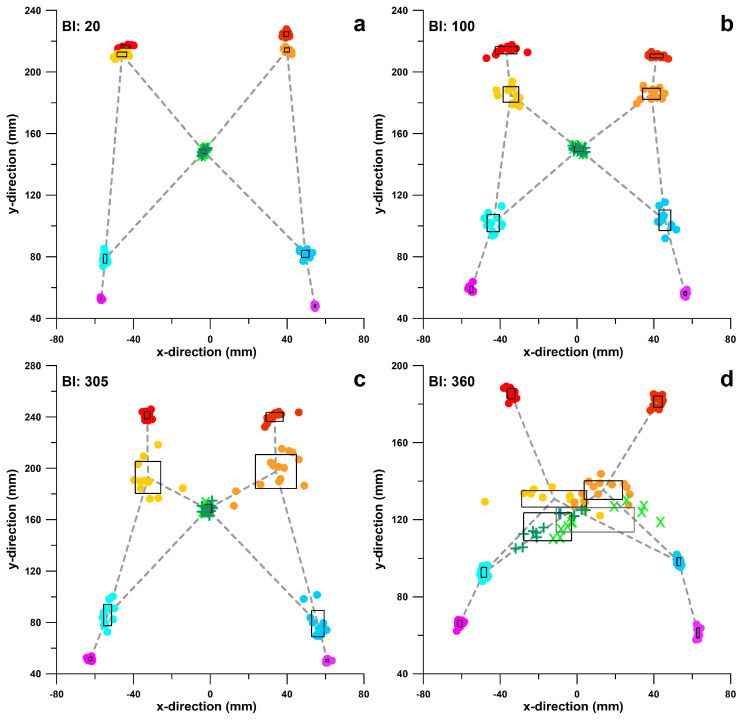
Clusters of the ten COP data points and the cyclogram for balance indices (BI) of 20 (**a**), 100 (**b**), 305 (**c**) and 360 (**d**); the black rectangles are centred at the average data of the scattered markers (*A*–*E*); the side lengths of the black rectangles correspond to 2 standard deviations of the scatter; clusters of zone *A* (cf. Figure 2c) are shown in red; clusters of zone *B* are shown in yellow/orange; clusters of zone *C* are shown in green; clusters of zone *D* are shown in blue; clusters of zone *D* are shown in magenta/purple; x: COP intersection point of cluster *C* at the weight shift from left to right in light green; **+**: COP intersection point of cluster *C* at the weight shift from right to left in dark green.

**Figure 4:**
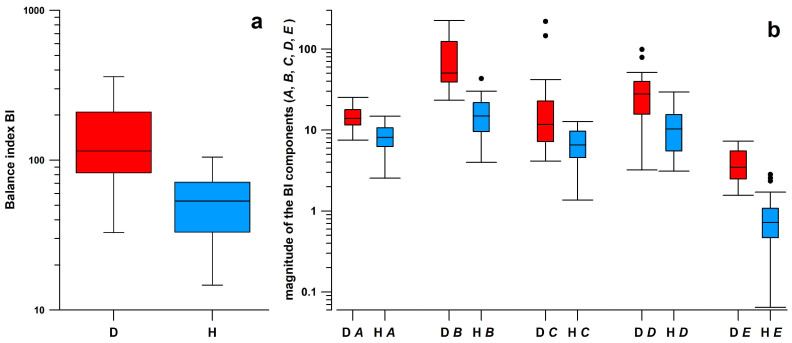
Box and whisker plots of (**a**) balance index (BI) and (**b**) magnitude of the 5 BI components (*A*,*B*,*C*,*D*,*E*); D = diabetes cohort, H = healthy cohort; all differences between H and D medians are highly significant (Table 2).

From the 10 points presented in Figure 2, we extracted 15 parameters to investigate how well they perform in separating the cohorts with diabetes (D) and healthy (H) individuals using an optimal threshold:

(1) The length of the gait line, i.e., the distance *AE* (Figure 2c);
(2) The length of the single-support line, i.e., the distance *BD* (Figure 2c);
(3) The anterior–posterior position of the COP intersection point (point *C*; Figure 2);
(4) The lateral position of point *C*;
(5) The anterior–posterior position of point *A* (Figure 2c);
(6) The anterior–posterior position of point *B* (Figure 2c);
(7) The anterior–posterior position of point *D* (Figure 2c);
(8) The anterior–posterior position of point *E* (Figure 2c);
(9) The length of distance *AB* (Figure 2c),
(10) The length of distance *BC* (Figure 2c),
(11) The length of distance *CD* (Figure 2c),
(12) The length of distance *DE* (Figure 2c),
(13) The ratio *Ra* of distance *BC* to distance *AB*;
(14) The ratio *Rp* of distance *CD* to distance *DE*; and
(15) A balance index BI, based on the hypothesis that the diabetes cohort diagnosed with peripheral neuropathy is less balanced than healthy volunteers.

The first 10 parameters refer to the geometry of the cyclogram, including the gait line. The first 4 parameters are standard measures included in software packages that assess the COP movements (e.g., Zebris; Isny, Germany). Parameters 5–8 correspond to the positions (x- and y-coordinates) of points *A*, *B*, *D*, and *E* (point *C* is included in the standard measures). Parameters 9–12 are the distances between the points (distances *AE* and *BD* are included in the standard measures). The lateral variability (another standard measure), defined as the standard deviation of *C* (lateral symmetry), is included in the BI.

The balance index (BI) was calculated based on the scatter of each of the 10 data clusters (Figure 2c). After calculating the standard deviations (σ) of COPx and COPy data for each cluster, the product of σ_x_ and σ_y_ was used as a measure of the scattering area for each cluster (Figure 2c). Corresponding left and right scattering areas were summed to reduce the number of areas to five (*A*–*E*; Figure 2c). The area of the red rectangles shown in Figure 2c corresponds to 2σ_x_·2σ_y_. The BI is the sum of the 5 scattering areas. The BI was evaluated for differences between the two cohorts (D and H). Since the original COP data were normalized to the sizes of the Pedar insoles, the raw BI was multiplied by a constant so that the optimal threshold was at BI = 100. Furthermore, we evaluated the individual contribution of each of the 5 scattering areas (*A*–*E*) to the BI.

Sensitivity and specificity of the 15 parameters were determined from the Mann–Whitney-U test and the ROC curve (receiver operating characteristic curve). The area under the ROC curve (AUC) corresponds to the Mann–Whitney U-statistic [32]. The Mann–Whitney-U test detected significant differences (*p* < 0.05) between healthy and diabetic cohorts. If the constant *S* is equal to U/(*n*_1_·*n*_2_), where *n*_1_ and *n*_2_ denote the number of data points compared in the Mann–Whitney test, U is the U-statistic, and U < 0.5 *n*_1_·*n*_2,_ then the effect size *r* is equal to 1—2*S*, and the AUC is equal to 1—*S*. The AUC represents the probability that, from a randomly selected positive and negative example, the positive is rated higher than the negative. The AUC ranges from 0.5 to 1, where AUC = 1 means that the two datasets are completely separate and do not overlap, and AUC = 0.5 results in a random classification. In addition, we identified the optimal discrimination thresholds. To achieve this, based on a range of different thresholds, we calculated the amount of data in a given dataset that were correctly (true positive, TP) and falsely (false positive, FP) identified as members of the diabetes D cohort (incorrectly because they belong to the healthy H cohort) by a specific threshold. Dividing TP by the actual number of positive cases (D), and FP by the actual number of negative cases (H) yields the true positive rate (TPR, sensitivity) and false positive rate (FPR = 1 − specificity). The optimal threshold is associated with the maximum value of √ (TPR·TNR), where TNR denotes the specificity. If the effect size was large (*r* > 0.37 [33]; AUC > 0.685), we determined the optimal threshold. In addition, we calculated TP (true positive) and FN (false negative) as a percentage of the actual number of positive cases (D) and TN (true negative) and FP (false positive) as a percentage of the actual number of negative cases (H).

Finally, we correlated the first 14 parameters with the 15th parameter (BI) to evaluate the dependencies of two fundamentally different concepts: medians of the positions of points on the cyclogram and lengths of distances and their ratios are related to the walking style, while the BI (derived from the standard deviations of the positions of points) expresses the state of balance.

## 3. Results

Figure 3 shows the cyclograms for BI data of 20, 100, 305, and 360. As the BI increases, the COP data points are more scattered, predominantly in the foremost points of the cyclogram (yellow and orange points in Figure 3), and the cyclogram collapses more, particularly evident in the decrease in distance *BC* (Figure 3; *BC* defined in Figure 2c).

### 3.1. Balance Index BI

The BI medians of the cohort with diabetes (D) and healthy (H) subjects differed significantly (Table 2; Figure 4), confirming, as expected, that diabetics with polyneuropathy are less balanced.

Of the individual clusters of the cyclogram (*A*–*E*), *B* contributes the most to the BI, followed by *D* (midstance clusters; Table 2). This result holds for both the percentual and the absolute contribution. Regarding the magnitude of the individual BI components, the mean values of D (red) and H cohorts (blue) differ significantly, and are consistently higher in the D cohort (Figure 4). Regarding the percentual contribution, all but one of the medians of D and H cohorts differ significantly (except for point *D*; Table 2).

### 3.2. Sensitivity and Specificity of COP Parameters

**Figure 5:**
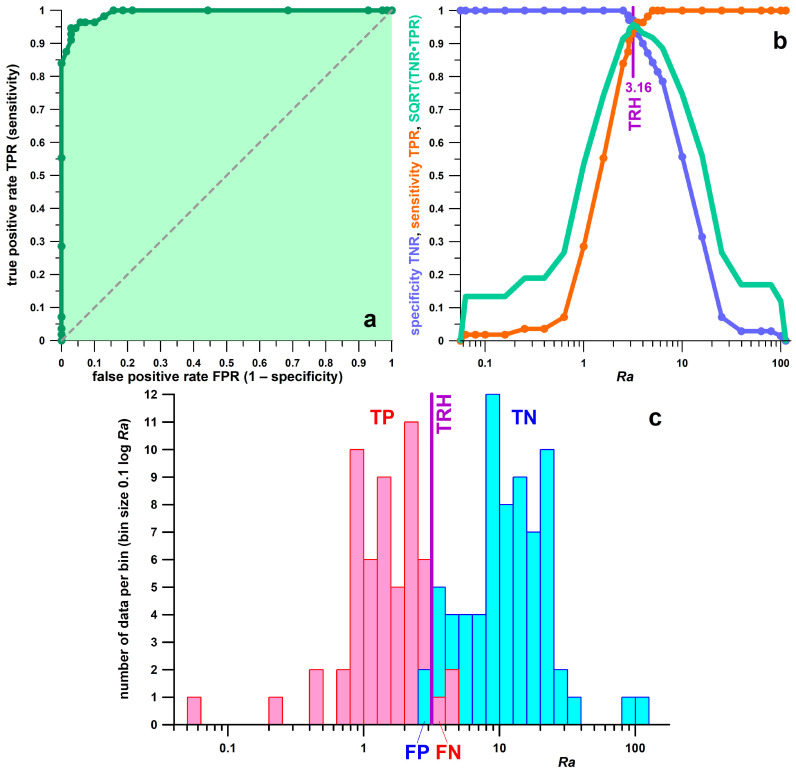
ROC curve (receiver operating characteristic) statistics of the parameter *Ra* (ratio of distance *BC* to distance *AB*; Figure 2c); (**a**) true positive rate (TPR, sensitivity) vs. false positive rate (FPR; 1 − specificity); the light green area corresponds to the AUC (area under the ROC curve; AUC = 0.993); the dotted line represents a random classifier (AUC = 0.5); (**b**) specificity (blue; TNR = true negative rate) and sensitivity (orange; TPR) vs. a range of *Ra* thresholds; the green curve is the square root of the product of TPR and TNR; the peak of the green curve represents the optimal threshold at maximum √ (TNR·TPR), which is related to Youden’s J index (J = TNR + TPR − 1; 0 ≤ J ≤ 1; at AUC = 1, TNR·TPR = TNR + TPR − 1); TRH: optimal threshold at 3.16; (**c**) histograms of the diabetes (D) cohort (red) and the healthy (H) cohort (blue) vs. the *Ra* parameter; TP = true positive (D); FN = false negative (D); TN = true negative (H); FP = false positive (H).

Table 3 explains how well the 15 parameters work as binary classifiers to predict positive (diabetes, D) and negative (healthy, H) cases from COP measurement results. The hierarchical decision criteria are as follows: significant difference (*p* < 0.05) between D and H medians; large effect size (r > 0.37); AUC close to 1 (perfect classifier if AUC = 1); and FN and FP as small as possible (preferably zero for a perfect classifier). These exclusion criteria left two candidates for optimal classifiers:

- Distance *AB*: AUC = 0.987; FN% = 1.79%; FP% = 7.14%;
- Ratio *Ra* (= *BC*/*AB*): AUC = 0.993; FN% = 5.36%; FP% = 2.86% (Figure 5).

Two other parameters had a similarly low FP percentage of 2.86%: *BC* and BI. However, their corresponding FN percentages were relatively high, with 12.50% and 32.14%, respectively.

The worst classifiers were as follows:

- Distance *DE*: AUC = 0.724; FN% = 32.14%; FP% = 30.00%;
- Distance *BD*: AUC = 0.724; FN% = 46.43%; FP% = 15.71%; and
- Distance *AE*: AUC = 0.750; FN% = 35.71%; FP% = 17.14%.

Unfeasible classifiers with a small AUC were the anterior–posterior position of *C*, the lateral position of *C*, and the anterior–posterior position of *A*.

Figure 5a shows the ROC curve for *Ra*, with the highest AUC of 0.993. The optimal threshold at the maximum AUC was 3.16 and was determined from the square root of the product of TPR and FPR (Figure 5b). The histograms in Figure 5c show the distribution of the *Ra* parameter for the D and H cohorts and the number of participants in whom positive (FP) or negative (FN) outcomes were falsely predicted.

### 3.3. Correlations Between BI and Other Parameters

**Figure 6:**
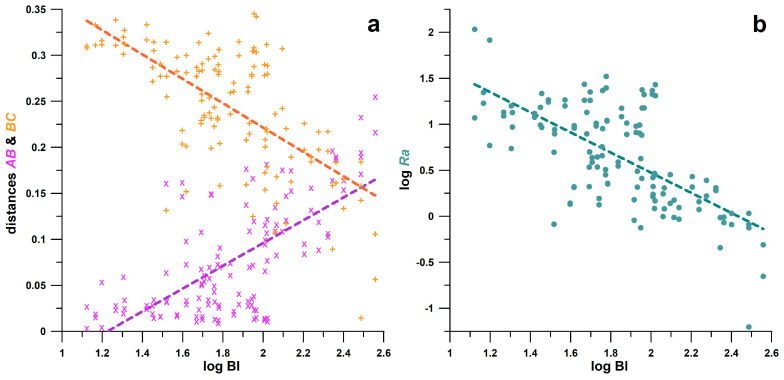
Correlations of the logarithm of the balance index (BI) with (**a**) the distances *AB* (×) and *BC* (+) and (**b**) the logarithm of ratio *Ra* (= *BC*/*AB*).

Figure 6 shows the three best correlations of BI with parameters *AB* (R^2^ = 0.4601, *p* < 0.0001), *Ra* (R^2^ = 0.4466, *p* < 0.0001) and *BC* (R^2^ = 0.4420, *p* < 0.0001). This result shows that the BI explains about 45% of the variation in *AB*, *Ra*, and *BC*, and vice versa, due to what these parameters have in common: different DPN levels, from no DPN to severe DPN. If the distances *AB* and *BC* and their ratio *Ra* are defined as independent variables, the proportion of the BI not explained by the independent variables is approximately 55%. It is likely that this proportion is due to the influence of age.

## 4. Discussion

The aim of this study was to extend existing cyclogram parameters to gain a more detailed insight into cyclogram dynamics. Conventional cyclogram measurements often appear to be used as provided by the software or applied by other authors (Table 1). There is limited research on new and innovative metrics, with two exceptions: Wong et al. [22,23] focused on pattern recognition, while Agrawal et al. [26] investigated cyclogram markers other than that of the intersection point.

The BI parameter uses the principle of the variability (standard deviation) of the COP intersection points (*C*). It is unclear why four of the six standard cyclogram parameters cover only point *C* (in terms of antero-posterior and lateral location and variability). The reason for this is probably that point *C* was used to assess symmetrical gait. Of five possible cyclogram markers (*A*–*E*), point *C* makes the second smallest contribution to the BI (Table 2). Surprisingly, only the variability of point *C* is included in the standard cyclogram measurements [8,9,10]. Nevertheless, the BI component of point *C* is significantly different in the H and D cohorts. This also applies to the other four points (*A*, *B*, *D*, and *E*) as well as to the BI as a whole: diabetics with DPN are, on average, less balanced than healthy people. In contrast to significant differences in the BI, the differences in the location of point *C* (antero-posterior and lateral) are not significant (Table 3). The lack of difference in the anteroposterior direction contradicts the results of Cao [27], who found a significant difference of 3.2–3.6 mm.

The ROC analysis (sensitivity and specificity; Table 3) revealed that a highly significant (*p* < 0.0001) difference between two medians with a large effect size does not necessarily represent a good classifier, even if the effect size is proportional to the AUC. For example, the single-support line (distance *BD*; Figure 1 and Figure 2) with an AUC of 0.724 has a sensitivity of 53.57% and false negatives of 46.43%. For the diabetes (D) cohort, *BD* is almost a random classifier.

In a clinical context, sensitivity and specificity are defined as follows:

Sensitivity represents the probability of a positive test result assuming the patient is affected by the disease (in the present study: diabetic polyneuropathy, DPN). “*When a sign, test or symptom has a high **sensitivity**, a **negative** result **rules out** the diagnosis*” [34].

Specificity represents the probability of a negative result assuming the patient is not affected by the disease. “*When a sign, test or symptom has an extremely high **specificity** …, a **positive** result tends to **rule in** the diagnosis*” [34].

The most sensitive classifiers (nintTP% ≥ 95%, where ‘nint’ is the nearest integer function) were the distance *AB* (98.21%) and the ratio *Ra* (94.64%; Table 3).

The most specific classifiers (nintTN% ≥ 95%) were *BC*, *Ra*, and the BI, with 97.14% (Table 3). Of these classifiers, we recommend *Ra* as the preferred classifier because of the following reasons:

- *Ra* is the only parameter with both high sensitivity and high specificity;
- *Ra* does not depend on the magnitude of the actual distances or on units, since *Ra* is a ratio of two distances (= *BC*/*AB*), and therefore is more convenient to implement as a classifier.

As mentioned in the introduction, the 10 g monofilament test is the most common diagnostic method to detect the loss of sensory function at the plantar surface. Two different studies showed that the 10 g monofilament test has a sensitivity of 0.60 [35] and 0.53 [36], and a specificity of 0.82 [35] and 0.88 [36]. The 10 g monofilament test therefore has a high probability of generating false negative test results (40–47%) in the diagnosis of DPN [37]. In both literature sources [35,36], nerve conduction studies were the reference standard for DPN, which is too time-consuming and expensive for routine clinical screening [36]. In contrast, since smart insoles should be available in diabetes and podiatry clinics for the reasons explained below, the results of the present study show that the *Ra* parameter with an AUC of 0.993 produced only 5.36% false negative and 2.86% false positive results.

The Sudoscan (Impeto Medical, Issy Les Moulineaux, France) assesses the sudomotor function by measuring the electrochemical skin conductance (ESC) of the hands and feet. ESC is used to detect cardiovascular autonomic neuropathy (CAN) for type 1 and type 2 diabetes and the CAN risk score [38]. To clarify, the two main forms of diabetic neuropathy are distal symmetric polyneuropathy (DPN) and diabetic autonomic neuropathies, particularly cardiovascular autonomic neuropathy (CAN) [7]. An ROC analysis of the CAN risk score to correctly classify CAN showed a sensitivity of 65% and specificity of 80%. (AUC of 0.75) [38]. While the monofilament test [7] and the Sudoscan [38] assess the somatosensory and visceromotor function of the peripheral nerves, respectively, a comprehensive and robust somatomotor function test that assesses the impact of lack of sensory input on cerebellar motor coordination is wanting. A walking test with smart insoles could fill this gap. This is all the more true since motor function in DPN is impaired through two different pathways:

- Afferent or sensory nerves fibres that transmit the inputs required for cerebellar motion coordination;
- Efferent or motor nerves that control the innervation of muscles.

In the latter case, the α-motor neuron is affected by reduced axional transport, decreased nerve conduction velocity, loss of axo-glial junctions, segmental demyelination, denervation of muscle fibres, and a reduced number of synaptic vesicles [39].

Although high-quality smart insoles with good resolution are still considered expensive laboratory equipment, the availability of smart insoles in diabetes care is expected as they are of paramount importance for testing the offloading potential of orthoses for the treatment of diabetic ulcers. To measure the pressure-relieving effect of orthoses, patients with diabetic ulcers are required to walk with pressure-sensitive insoles. The pressure data collected during this process can be converted into forces and positions of the COP. From the COP data, the cyclogram parameters are calculated and can be used to diagnose the severity of DPN. Therefore, cyclogram parameters can be collected at each appointment of a diabetic patient, provided that smart insoles are available.

The disadvantage of smart insoles is that the results of the cyclogram are not specific, as they respond to any type of balance disturbance caused by, for example, vestibular, cardiovascular, skeletal, visual, medicinal, traumatic, or age-related disorders, to name a few. The same problems apply to the Sudoscan, which responds to any other disturbance of sudomotor function such as compression or disruption of peripheral nerves (also detected by Moberg’s ninhydrin test [40]). In addition, the smart insoles themselves may also affect gait because they are 2 mm thick and have limited flexibility as they do not conform to 3D surfaces. If patients wear orthopaedic insoles, the smart insoles should be placed underneath them, provided they are not too stiff or too thick. The positions of the cyclogram markers (points *A*–*E*) must be normalized to the size of the smart insoles, with the length of the smart insole equal to 1 or 100% at any given size. The distances between the points are calculated from the normalized data.

The limitations of this study are twofold:

First, the two cohorts (diabetes and healthy) were not age-matched, because it was impossible to find the required number of younger T2D individuals with DPN. We might therefore expect that age-related and DPN-related gait disturbances would be intermingled in the diabetes cohort. However, the use of age-mismatched cohorts is not uncommon. For example, Gouelle et al. [41] compared 123 healthy subjects (aged 12–65 years) with 31 patients (aged 12–25 years) with Friedreich’s ataxia (FRDA) and compared the gait index in FRDA with that of the healthy population.

Second, the two cohorts (diabetes and healthy) could be considered too fundamentally different, since the diabetic patients were severe cases of DPN. The hypothesis that the diabetes cohort diagnosed with peripheral neuropathy is less balanced than healthy volunteers was therefore confirmed by the balance index, BI. Although the monofilament test was not used as a diagnostic gold standard (which, as outlined above, it is not), it was expected that the patients would pass the monofilament test positively.

However, and this also applies to the first limitation, this study did not focus specifically on the balance index, but rather aimed to investigate other cyclogram parameters, such as positions of cyclogram markers, distances between the markers, and the ratios of these distances. While the standard deviations and hence inconsistencies of the markers (points *A*–*E*) are expected to correlate with the severity of balance problems, the far less inconsistent (and hence highly significant) distances and their ratios between the diabetes and healthy cohorts are not easily explained in terms of balance. Figure 6 clearly shows that 55% of the BI cannot be explained by *AB*, *BC*, and *Ra*, and this proportion is therefore likely due to age-related balance problems. Theoretically, it could very well be that the changing geometry of the cyclogram outlined in Figure 3 represents DPN-specific, even combined with diabetic foot-specific, changes in gait.

Regarding this connection, future research should focus on cyclogram patterns, linking them to accurately diagnosed diseases through small clinical trials with age-matched cohorts, followed by multicentre studies, combining various metrics (beyond cyclograms) to deepen our understanding of cyclogram dynamics. In addition, new metrics should be implemented in existing software packages to promote innovation in the diagnosis of DPN.

## 5. Conclusions

The main findings of this study were that healthy volunteers and diabetic subjects had significantly different cyclogram geometries (point markers and distances between these points) and BI indices. Two distances and their ratio with an AUC > 0.94 had the potential to serve as classifiers with very high sensitivity and specificity for the diagnosis of DPN. The recommendations arising from these results are that future research should include larger and age-matched cohorts of healthy and diabetic subjects, preferably with multicentre studies, and that the new parameters tested in this present study should be implemented in the software of existing technology capable of measuring cyclograms.

## Figures and Tables

**Table 1:** Literature overview of cyclogram applications, related to the underlying measurement technology, medical conditions, and applied cyclogram metric; ap pos: antero-posterior position of the COP intersection points; ap var: antero-posterior variability (standard deviation) of the COP intersection points, lat pos: lateral position of the COP intersection points, lat var: lateral variability (standard deviation) of the COP intersection points.

Reference	Technology	Model	Medical Condition	Conditions Compared	Cyclogram Metric
Kalron et al. [11]	treadmill	Zebris	multiple sclerosis	multiple sclerosis vs. healthy	ap var, lat pos, lat var
Kalron and Frid [12]	treadmill	Zebris	multiple sclerosis	cerebellar function, non/slight vs. mild/moderate disability	ap var, lat pos, lat var
Cha and Park [13]	treadmill	Zebris	low back pain	correlation (e.g., with age)	ap var, lat pos
Lee and Liang [14]	treadmill	Bertec	stroke	stroke vs. healthy	ap pos
Cao et al. [15]	treadmill	Zebris	above knee amputation with prosthesis	none	gait line
Shin and Ahn [16]	treadmill	Zebris	Parkinson	Parkinson vs. healthy	gait line, midstance line, ap var, lat pos, lat var
Ichimura [17]	treadmill	Tec Gihan	above knee amputation with prosthesis	amputation vs. healthy	ap var, lat pos, lat var
Padula et al. [18]	pressure mat	Zebris	concussions, stroke, Lyme disease	“before vs. after intervention with yoked prisms”	ap pos, lat pos
Gimunová et al. [19]	pressure mat	Zebris	alcohol intoxication	intoxicated vs. sober	gait line, ap pos, ap var, lat pos, lat var
Datta-Gupta et al. [20]	pressure mat	GaitRite	Parkinson, foot dystonia	before vs. after injection of botulinum toxin	none; cyclogram shown by example
Chen et al. [21]	smart shoe	7 strain-gauged force transducers (DIY)	stroke	stroke vs. healthy	none; qualitative comparison of cyclograms
Wong et al. [22]	smart shoe	8 force sensors, Infotronic Computer Dyno Graphy instrumented ‘overshoe’	cerebral palsy	botulinum toxin injection vs. phenol injection vs. normal groups	“gait line pattern”, “cyclogram pattern”
Wong et al. [23]	smart shoe	8 force sensors, Infotronic Computer Dyno Graphy instrumented ‘overshoe’	stroke	stroke vs. healthy	“gait line pattern”, “cyclogram pattern”
Pfaffen et al. [24]	smart insole	16 piezo-resistive sensors (DIY)	none	none	none; cyclogram shown by example
Chou et al. [25]	smart insole	89 piezo-resistive sensors (Industrial Technology Research Institute, Taiwan)	none	none	ap pos, lat pos
Agrawal et al. [26]	smart insole	5 piezo-resistive sensors (Suratec, Thailand)	fall risk	non-fall vs. fall	single-support line, other distances between cyclogram markers
Cao et al. [27]	smart insole	Medilogic	diabetes	diabetes (D) with polyneuropathy (DPN) + peripheral arterial disease (PAD) vs. D with DPN vs. D with PAD vs. D w/o DPN/PAD vs. healthy	ap pos
Stramel et al. [28]	smart insole	3 piezo-resistive sensors (DIY)	stroke	none	single-support line, ap pos, lat pos
Chu et al. [29]	not reported	not reported	Parkinson	before vs. after intervention (spinal manipulation, gait training)	ap pos, lat pos

**Table 2:** Statistics of the standard deviations of the COP markers (*A*–*E*, and their sum, the BI shown in Figure 2c); D = diabetes cohort, H = healthy cohort; BI = balance index; *p* = *p*-value; r = effect size.

Parameter	Median D	Median H	*p*	r	Effect Size
*Balance Index* BI					
BI	115.5	53.3	<0.001	0.703	large
*Contribution of A, B, C, D, E to* BI (%)					
*A*	8.6	19.5	<0.0001	0.643	large
*B*	48.1	36.0	0.0007	0.500	large
*C*	8.7	14.7	0.0168	0.353	medium
*D*	23.3	25.5	0.2263	0.180	small
*E*	2.7	1.4	0.0065	0.402	large
*Magnitude of the* BI *components* (*A, B, C, D, E*)					
*A*	13.9	8.1	<0.0001	0.727	large
*B*	50.5	14.8	<0.0001	0.965	large
*C*	11.7	6.6	0.0002	0.551	large
*D*	27.8	10.3	<0.0001	0.700	large
*E*	3.5	0.7	<0.0001	0.951	large

**Table 3:** Comparison of medians of cyclogram parameters suitable as potential classifiers, and the corresponding ROC curve (receiver operating characteristic) statistics; COP markers *A*–*E* and the distances between the markers are explained in Figure 2c; ap: antero-posterior position of a marker; lat: lateral position of a marker; *Ra*: ratio of distance *BC* to distance *AB*; *Rp*: ratio of distance *CD* to distance *DE*; BI = balance index; D = diabetes cohort, H = healthy cohort; *p* = *p*-value; r = effect size; AUC: area under the ROC curve; THR: optimal threshold at maximum AUC; SE = sensitivity; SP = specificity; TP%: percentage of true positives; FN%: percentage of false negatives; TN%: percentage of true negatives; FP%: percentage of false positives; columns r, AUC, FN%, FP% are colour-coded from red (worst result) to green (best result); for very small and small effect sizes, the data of the last 5 columns are not shown, as the corresponding parameters do not qualify as classifiers.

No.	Parameter	Median D	Median H	*p*	*r*	Effect Size	AUC	TRH	TP% (D), SE	FN% (D)	TN% (H), SP	FP% (H)
1	*AE*	0.419	0.465	<0.0001	0.501	large	0.750	0.440	64.29	35.71	82.86	17.14
2	*BD*	0.210	0.203	<0.0001	0.447	large	0.724	0.212	53.57	46.43	84.29	15.71
3	ap *C*	0.557	0.564	0.3576	0.096	v-sm	0.548					
4	lat *C*	−0.004	−0.003	0.4839	0.073	v-sm	0.536					
5	ap *A*	0.805	0.833	0.0244	0.234	small	0.617					
6	ap *B*	0.695	0.810	<0.0001	0.749	large	0.875	0.746	75.00	25.00	85.71	14.29
7	ap *D*	0.377	0.306	<0.0001	0.676	large	0.838	0.328	80.36	19.64	74.29	25.71
8	ap *E*	0.008	0.004	<0.0001	0.669	large	0.834	0.005	83.93	16.07	74.29	25.71
9	*AB*	0.126	0.027	<0.0001	0.974	large	0.987	0.065	98.21	1.79	92.86	7.14
10	*BC*	0.193	0.288	<0.0001	0.883	large	0.941	0.231	87.50	12.50	97.14	2.86
11	*CD*	0.265	0.322	<0.0001	0.669	large	0.834	0.290	73.21	26.79	90	10.00
12	*DE*	0.150	0.120	<0.0001	0.447	large	0.724	0.135	67.86	32.14	70	30.00
13	*Ra*	1.487	11.365	<0.0001	0.985	large	0.993	3.160	94.64	5.36	97.14	2.86
14	*Rp*	1.729	2.737	<0.0001	0.609	large	0.805	2.239	73.21	26.79	74.29	25.71
15	BI	115.5	53.300	<0.0001	0.703	large	0.852	100.0	67.86	32.14	97.14	2.86

## Data Availability

The data presented in this study are available on request from the first author to any qualified researcher who has obtained Ethics Approval for secondary use of existing data through a Consent Waiver.
